# Does information on novel identified autoantibodies contribute to predicting the progression from undifferentiated arthritis to rheumatoid arthritis: a study on anti-CarP antibodies as an example

**DOI:** 10.1186/s13075-018-1591-2

**Published:** 2018-05-03

**Authors:** Debbie M. Boeters, Leendert A. Trouw, Annette H. M. van der Helm-van Mil, Hanna W. van Steenbergen

**Affiliations:** 1 Department of Rheumatology, Leiden University Medical Center, P.O. Box 9600, 2300 RC Leiden, the Netherlands; 2 Department of Immunohematology and Blood Transfusion, Leiden University Medical Center, Leiden, The Netherlands; 3 Department of Rheumatology, Erasmus University Medical Center, Rotterdam, the Netherlands

**Keywords:** Rheumatoid arthritis, Anti-CarP antibodies, 2010 classification criteria

## Abstract

**Background:**

The presence of autoantibodies is considered an important characteristic of rheumatoid arthritis (RA); therefore, both anticitrullinated protein antibodies (ACPA) and rheumatoid factor (RF) are included in the 2010 classification criteria for rheumatoid arthritis (RA). However, a considerable number of RA patients lack both these autoantibodies. Recently, several novel autoantibodies have been identified but their value for the classification of RA patients is unclear. Therefore, we studied the value of novel autoantibodies using the presence of anticarbamylated protein (anti-CarP) antibodies as an example for predicting RA development in patients with undifferentiated arthritis (UA).

**Methods:**

There were 1352 UA patients included in the Leiden Early Arthritis Clinic (EAC) cohort according to the 1987 criteria. When the 2010 criteria were used, there were 838 UA patients. Of these, we evaluated whether they fulfilled the 1987 or 2010 criteria after 1 year, respectively. Logistic regression analyses were performed with RA as outcome and ACPA, RF, and anti-CarP antibodies as predictors. Analyses were repeated after stratification for ACPA and RF.

**Results:**

Thirty-three percent of the 1987-UA patients and 6% of the 2010-UA patients progressed to RA during the first year of follow-up. For the 1987-UA patients, anti-CarP antibodies were associated with progression to RA, an association which remained when a correction was made for the presence of ACPA and RF (odds ratio (OR) 1.7, 95% confidence interval (CI) 1.2–2.4). After stratification for ACPA and RF, anti-CarP antibodies were associated with progression to RA only for ACPA- and RF-negative patients (OR 2.1, 95% CI 1.3–3.7). For the 2010-UA patients, anti-CarP antibodies were associated with progression to RA; however, they were not when a correction was made for the presence of ACPA and RF (OR 0.8, 95% CI 0.3–2.1).

**Conclusions:**

Our finding that anti-CarP antibodies have no additional value when RA is defined according to the 2010 criteria might be inherent to the composition of the 2010 criteria and therefore might also apply to other novel autoantibodies. Potentially it would be interesting to evaluate other, non-autoantibody biomarkers.

## Background

Rheumatoid arthritis (RA) is characterized by the presence of autoantibodies, the most characteristic among which are anticitrullinated protein antibodies (ACPA) and rheumatoid factor (RF). These are used as diagnostic tools and are included in the classification criteria for RA [1]. Nonetheless, in approximately one-third of early RA patients these autoantibodies are lacking [2]. It is important to better characterize these patients since early intervention in seronegative RA is also important. Therefore, research has focused on identifying novel autoantibodies and several have been identified [3–7]. Based on this research, two issues have been raised. First, stratified analyses are pivotal to prove an additive value of a test. A novel autoantibody should predict an outcome in patients negative for both ACPA and RF, or in patient groups with a similar presence of ACPA and/or RF (e.g., ACPA+RF+novel autoantibody+ vsACPA+RF+novel autoantibody- patients). Thus far, studies that have evaluated the predictive value of novel autoantibodies are often stratified for ACPA but not for RF, leaving the question unanswered if the findings attributed to the novel autoantibody were actually driven by the concomitant presence of RF [5, 8]. A second issue is that, although different disease stages of RA have been studied, the value of novel autoantibodies in identifying the patients that will develop RA among patients presenting with undifferentiated arthritis (UA) is undetermined. Only one study evaluated the role of novel autoantibodies (UH-RA.1, UH-RA.21) in UA patients as an early marker of RA development [4]. The ultimate aim of this study was to increase our understanding on the value of recently identified autoantibodies to predict RA development using accurate stratification for ACPA and RF. An interesting novel family of autoantibodies are the anticarbamylated protein (anti-CarP) antibodies which target proteins modified by carbamylation. These antibodies are present in RA patients and are associated with the severity of radiographic progression [7, 9]. In this study, we investigated the value of the novel anti-CarP antibodies in predicting RA development in patients with UA, independent of ACPA and RF [7].

## Methods

### Patients

Between 1993 and 2015, 1352 UA patients (according to the 1987 criteria; 1987-UA) were included in the Leiden Early Arthritis Clinic (EAC) cohort. This became 838 UA patients when the 2010 criteria were used (2010-UA). The EAC is an inception cohort that was started in 1993 and includes patients with clinically confirmed arthritis with symptom duration < 2 years at presentation to the rheumatologist [10]. Baseline questionnaires, joint counts, and blood samples were collected, and radiographs were taken. Two weeks after inclusion, when the results of laboratory investigations and radiography were known, patients received their diagnosis. Classification criteria were only applied to patients with a clinical diagnosis or suspicion of RA, and patients who were not classified according to RA classification criteria were documented as having UA.

### Anti-CCP2, RF, and anti-CarP measurements

Baseline serum samples were tested for ACPA, RF, and anti-CarP antibodies. Immunoglobulin (Ig)G antibodies to cyclic citrullinated peptide (CCP) were measured by second generation anti-CCP2 enzyme-linked immunosorbent assay (ELISA; Immunoscan RA Mark 2, Eurodiagnostica, Arnhem; cut-off 25 U/ml), and anti-CCP2 ELISA (EliA CCP, Phadia, Nieuwegein, the Netherlands; cut-off 7 U/ml). IgM RF was determined by an in-house ELISA. IgG anti-CarP antibodies were determined as described previously in the Leiden EAC [7]. As no commercial kit is available for anti-CarP antibodies, we used our own in-house anti-CarP assay based on carbamylated fetal calf serum and, as a control, nonmodified fetal calf serum as the coating antigens in the ELISA. Cut-off for positivity was based on the mean + 2 standard deviations (SDs) from a set of healthy controls.

### Analyses

Analyses were first performed when RA was classified using the 1987 criteria; thereafter, analyses were repeated using the 2010 criteria since autoantibodies are more prominent in the 2010 criteria. Fulfilment of the 1987 criteria and 2010 criteria was evaluated after 1 year of follow-up for the 1987-UA and 2010-UA patients, respectively. Logistic regression analyses were performed with ACPA, RF, and anti-CarP antibodies as independent variables and RA as outcome, both in the total group of UA patients and after stratification for ACPA and RF status.

## Results

Baseline characteristics of the 1352 1987-UA and 838 2010-UA patients are shown in Table 1. Of these UA patients, 33% (441/1352) and 6% (53/838) progressed to RA during the first year according to the 1987 and 2010 criteria, respectively. Of the 1352 1987-UA patients, 257 (19%) were anti-CarP positive and of the 838 2010-UA patients, 77 (9%) were anti-CarP positive.

**Table 1:** Baseline characteristics of the total group of UA patients and the subgroups of patients with UA according to the 1987 and the 2010 criteria

	Total group of UA patients	Subgroup of 1987 UA patients	Subgroup of 2010 UA patients
	(*n* = 1430)	(*n* = 1352)	(*n* = 838)
Age (years), mean (SD)	53 (17)	53 (17)	51 (17)
Female, *n* (%)	882 (62)	837 (62)	494 (59)
Symptom duration (weeks), median (IQR)	14 (6-31)	14 (6-31)	12 (5-28)
66-SJC, median (IQR)	3 (1-7)	3 (1-7)	2 (1-4)
68-TJC, median (IQR)	4 (1-10)	4 (1-10)	2 (1-5)
CRP (mg/ml), median (IQR)	8 (3-22)	8 (3-22)	6 (3-19)
ACPA positivity, *n* (%)	297 (21)	283 (21)	48 (6)
RF positivity, *n* (%)	374 (26)	359 (27)	68 (8)
anti-CarP positivity, *n* (%)	271 (19)	257 (19)	77 (9)

Of the total group of UA patients (*n* = 1430), 760 patients have UA both according to the 1987 and 2010 criteria, 592 patients only have UA according to the 1987 criteria, and 78 patients only have UA according to the 2010 criteria

ACPA, anticitrullinated protein antibodies; anti-CarP, anticarbamylated protein antibodies; CRP, C-reactive protein; IQR, interquartile range; RF, rheumatoid factor; SD, standard deviation; SJC, swollen joint count; TJC, tender joint count; UA, undifferentiated arthritis; symptom duration, time between symptom onset and inclusion in cohort

The value of anti-CarP antibodies was first studied in the 1987-UA patients. The presence of anti-CarP antibodies at baseline was associated with progression to RA (odds ratio (OR) 4.2, 95% confidence interval (CI) 3.2–5.6), an association which remained when a correction was made for the presence of ACPA and RF (OR 1.7, 95% CI 1.2–2.4). There was no additional predictive value of anti-CarP antibody levels in anti-CarP-positive patients. The association of anti-CarP antibodies with progression to RA was then determined within the strata of patients with a similar ACPA and RF status. The majority of the UA patients (69%) were ACPA- and RF-negative; 7% (65/929) of these ACPA- and RF-negative patients had anti-CarP antibodies (Table 2). Within this subgroup, the presence of anti-CarP antibodies was statistically significantly associated with progression to RA (OR 2.1, 95% CI 1.3–3.7; Table 2). When absolute risks were examined, the pre-test risk for RA development in the ACPA- and RF-negative subgroup was 21%, and this increased to 35% when anti-CarP antibodies were present (Table 2). When exploring the negative predictive value (NPV), the pre-test risk of not developing RA was 79% which was similar to the NPV of 80%.

**Table 2:** Proportion of 2010-UA and 1987-UA patients progressing to RA within 1 year within groups of similar ACPA and RF status

		RA	No RA	% RA/% non-RA development (pre-test risks)^a^	OR (95% CI)	PPV (95% CI)	NPV (95% CI)
1987-UA patients (*n* = 1352)							
ACPA–RF– (*n* = 929)	anti-CarP+	23	42	21/79	2.1 (1.3–3.7)	35 (25–48)	80 (77–82)
	anti-CarP–	176	688				
ACPA+RF– (*n* = 64)	anti-CarP+	16	10	50/50	2.2 (0.8–6.1)	62 (43–78)	58 (42–72)
	anti-CarP–	16	22				
ACPA–RF+ (*n* = 140)	anti-CarP+	8	9	39/61	1.4 (0.5–4.0)	47 (26–69)	62 (53–70)
	anti-CarP–	47	76				
ACPA+RF+ (*n* = 219)	anti-CarP+	107	42	71/29	1.2 (0.6–2.2)	72 (64–78)	31 (22–43)
	anti-CarP–	48	22				
2010-UA patients (*n* = 838)							
ACPA–RF– (*n* = 755)	anti-CarP+	1	48	4/96	0.5 (0.1–3.5)	2 (0–11)	96 (94–97)
	anti-CarP–	30	676				
ACPA+RF– (*n* = 15)	anti-CarP+	1	2	13/87	5.5 (0.2–129)	33 (6–79)	92 (65–99)
	anti-CarP–	1	11				
ACPA–RF+ (*n* = 35)	anti-CarP+	0	4	17/83	Undefined	0 (0–49)	81 (64–91)
	anti-CarP–	6	25				
ACPA+RF+ (*n* = 33)	anti-CarP+	9	12	42/58	1.1 (0.2–4.4)	43 (24–63)	58 (32–81)
	anti-CarP–	5	7				

Patients were stratified according to the presence of different autoantibody combinations (ACPA–RF–, ACPA+RF–, ACPA–RF+, and ACPA+RF+); within these groups the predictive value of the presence of anticarbamylated protein (anti-CarP) antibodies for progression to rheumatoid arthritis (RA) was determined, both within 2010-undifferentiated arthritis (UA) and 1987-UA patients

ACPA, anticitrullinated protein antibodies; CI, confidence interval; NPV, negative predictive value; OR, odds ratio; PPV, positive predictive value; RF, rheumatoid factor

^a^ Observed risk of RA development within ACPA and RF strata (pre-test risks), without information on anti-CarP status

Next, the predictive value of anti-CarP antibodies was studied within the 2010-UA patients. Here, anti-CarP antibodies at baseline were associated with progression to 2010 RA within 1 year (OR 2.9, 95% CI 1.4–5.8). However, when adjustment was made for the presence of ACPA and RF, there was no additive predictive value of anti-CarP antibodies (OR 0.8, 95% CI 0.3–2.1). When analyzing groups of patients stratified according to the absence of ACPA and RF, the majority of 2010-UA patients were ACPA- and RF-negative (90%) and only 6% (49/755) of these patients had anti-CarP antibodies. Within this subgroup, no predictive value of anti-CarP antibodies was observed (Table 2). Evaluation of absolute risks in the ACPA- and RF-negative subgroup revealed that the pre-test risk of developing RA was 4% and the positive predictive value (PPV) was 2% when anti-CarP antibodies were present. Likewise, the pre-test risk of not developing RA in this subgroup was similar to the post-test risk (NPV) when patients tested negative for anti-CarP antibodies (both 96%, Table 2).

## Discussion

This study was performed to increase our understanding of the value of recently identified autoantibodies to predict RA development using accurate stratification for ACPA and RF. Anti-CarP antibodies were studied as an example. We observed that the presence of anti-CarP antibodies was statistically significantly associated with the development of RA within ACPA- and RF-negative 1987-UA patients. In this group, the risk of developing RA increased from 21% to 35% when anti-CarP antibodies were present. However, when RA was defined according to the 2010 criteria, anti-CarP antibodies were not associated with RA development and the presence of these autoantibodies did not increase the risk of RA development compared to the pre-test risks.

Although they used different study designs and entire early arthritis populations, two previous studies found 2.2% and 0.4% improved classification when adding anti-CarP to ACPA and RF, thus showing little additive benefit [8, 11]. These findings are in line with our data.

**Fig. 1:**
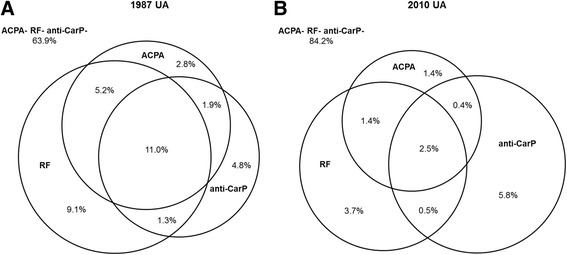
Concomitant presence of anticitrullinated protein antibodies (ACPA), rheumatoid factor (RF), and anticarbamylated protein (anti-CarP) antibodies in patients with 1987-undifferentiated arthritis (UA) and 2010-UA. Depicted are the percentages of the 1352 1987-UA (**a**) and the 838 2010-UA (**b**) patients positive for ACPA, RF, and/or anti-CarP antibodies

Presumably this finding is explained by the fact that ACPA and RF are heavily weighted in the 2010 criteria. Consequently, the majority of UA patients are ACPA- and RF-negative and these patients can only fulfill the 2010 criteria if they develop > 10 involved joints but they can fulfill the 1987 criteria over time with less extensive disease progression; hence the definition of the outcome matters. Additionally, autoantibodies frequently occur together (Fig. 1), as has been shown for several novel autoantibodies [3, 5]. These two explanations might also apply to other novel autoantibodies. Although novel autoantibodies other than anti-CarP antibodies were not evaluated here, we anticipate that similar findings will be obtained. Importantly, our findings relate to the earlier identification of patients with RA; novel autoantibodies can still be useful for other outcomes, such as radiographic progression [7].

## Conclusions

More research is needed to identify early RA patients among (2010 criteria-negative) UA patients, but based on the composition of the current classification criteria it will be interesting to evaluate other, non-autoantibody biomarkers.

## Abbreviations

ACPA: Anticitrullinated protein antibodies
anti-CarP: Anticarbamylated protein
CCP: Cyclic citrullinated peptide
CI: Confidence interval
EAC: Early Arthritis Clinic
ELISA: Enzyme-linked immunosorbent assay
Ig: Immunoglobulin
NPV: Negative predictive value
OR: Odds ratio
RA: Rheumatoid arthritis
RF: Rheumatoid factor
SD: Standard deviation
UA: Undifferentiated arthritis
